# Why do people with similar levels of internal control differ in their likelihood to commit fraud? Analysis of the moderating effect of perceived opportunity to commit fraud

**DOI:** 10.3389/fpsyg.2022.999469

**Published:** 2022-11-09

**Authors:** Xiaonan Sun, Yan Chen

**Affiliations:** ^1^Accounting Department, Dongbei University of Finance and Economics, Dalian, China; ^2^Accounting Department, Dalian University of Finance and Economics, Dalian, China

**Keywords:** disposition, self-efficacy, fraud opportunity, perception, decision making

## Abstract

This research focuses on one of the three components of the fraud triangle, namely opportunity, and empirically tests the tendency to commit fraud. The perceived opportunity to commit fraud can be considered in terms of temptation and obstacles. This research employs concepts from cognitive psychology, i.e., desire and motivation for action, which affect people’s decision-making processes when presented with an opportunity to commit fraud. Questionnaires were used to analyze the tendency to commit fraud. First, dispositional differences among people differing in self-control were observed, which may influence the likelihood of fraudulent behavior. That is, low self-control mediates the relationship between self-regard and fraud tendency. Second, strong relationships of both personal disposition and self-efficacy with the tendency to commit fraud were revealed; high self-efficacy enhances the tendency to commit fraud. This research offers a new psychological perspective on fraud opportunity, and showing for the first time that fraud can be reduced not only by improved internal control and external supervision, as suggested in previous studies, but also by changing people’s perceptions of fraud opportunity, given the fallibility of both internal control and external supervision.

## Introduction

Financial fraud has always been a serious problem, but has become especially problematic since the coronavirus 2019 (COVID-19) pandemic. According to the Association of Certified Fraud Examiners (ACFE; 2021), 51% of the surveyed organizations discovered more fraud since the onset of the COVID-19 pandemic and 71% expect the level of fraud to increase over the next year. Many studies on financial fraud focused on the fraud triangle, which is the classical theory of fraudulent behavior. The fraud triangle consists of three components: pressure, opportunity, and rationalization (Cressey, 1950, 1953). People may feel compelled to conduct fraud to alleviate perceived pressure, while opportunity refers to a situation in which it is assumed that fraudulent behavior will not be recognized or penalized. Rationalization refers to the justification of a moral transgression. Scholars and auditors use the fraud triangle as a conceptual model to explore the motivation and antecedents of fraud behavior (Dorminey et al., 2012). However, studies evoking the fraud triangle usually neglect the impact of personality traits and psychological Processes. Updates and revisions of the fraud triangle, to include human factors, have been proposed previously. To better prevent and detect financial fraud, Albrecht et al. (1984) added personal integrity to the fraud triangle, while Wolfe and Hermanson (2004) added personal capability, resulting in a fraud diamond. Choo and Tan (2007) also expanded the fraud triangle to derive their “Broken Trust” and “American Dream” theories. Dorminey et al. (2012) constructed the MICE (money, ideology, coercion and ego) model to analyze people’s motivations to commit fraud. Raval (2018) developed a disposition-based fraud model (DFM) as a modified fraud triangle, based on several psychological theories and concepts. All of these researchers either added to the fraud triangle or completely replaced its original components (Davis and Pesch, 2013; Morales et al., 2014; Ismail, 2019; Maulidi, 2020). As such, the fraud triangle has been somewhat diminished as an explanation of fraud due to the lack of consideration of human factors. However, careful review of the fraud triangle shows that this classical model still has explanatory power, although it is necessary to empirically test the effects of human factors on the tendency to commit fraud, especially on perceptions of the components of the triangle.

Specifically, fraud opportunity, as one component of the triangle, is usually considered to be an objective situation that may be grasped to commit fraud and without being caught. According to current international standards, fraud and corruption occur when necessary and reasonable organizational measures are not in place (Hauser and Hogenacker, 2014; Hauser, 2019). In US audit standards, the opportunity for fraud arises when there are lack of (or ineffective) controls, or the potential perpetrator has the capacity to bypass controls. Ineffective governance and control mechanisms such as weak internal control systems, company cultures that encourage unethical behavior, and a lack of monitoring, can foster corruption (Zahra et al., 2005; Pfarrer et al., 2008; Williams, 2013). Even in the field of criminology, opportunity is considered a prerequisite for fraud, as it reduces the likelihood of being caught; psychological factors are only associated with the rationalization, which comprises another part of the fraud triangle (Anand et al., 2004; Murphy and Dacin, 2011; Murphy, 2012). However, according to agency theory, individuals act in rational and self-interested ways; if the opportunity and pressure aspects of the fraud triangle are both fulfilled, fraud will take place (Cohen et al., 2007). A question arises as to whether there are specific psychological factors that affect the perceptions of the opportunity to commit fraud. The original fraud triangle defined fraud opportunity as of the perceived chance to achieve one’s goals or relieve pressure by secretly engaging in fraud (Cressey, 1953), which underscores the importance of psychological and cognitive processes. Previous studies suggested that internal control and outside supervision should be enhanced to prevent fraud, but did not tend to consider people’s decision-making processes. When different people have the same opportunity to commit fraud, what explains their different actions? Even within the same environment, and with the same level of internal control, some people commit fraud while others do not. This is necessarily attributable to differences in cognitive processes. Opportunity may in fact subsume objective conditions and psychological factors, where the latter may influence how people perceive, and behave in response to, fraud opportunities. Thus, the most objective and concrete factor of the fraud triangle, there is still mediated by psychological processes; this paper explores this hitherto neglected topic. We hope that, by applying psychological theories to explain people’s perceptions of fraud opportunity and related psychological processes, interventions to modify people’s perceptions of fraud opportunity may be developed to better curtail fraud behavior.

The remainder of this paper is organized as follows: in the next section, we review the relevant prior literature and theories, which informed our hypotheses. In Section III, we describe the research methodology, questionnaire design and sample characteristics. We present the empirical results and analysis in Section IV and, in Section V, summarize our major findings, and discuss the study limitations and directions for future research.

## Literature review and hypotheses

### Fraud opportunity

The most popular and classical fraud models are rooted in Cressey (1953) study, in which the preconditions for fraudulent behavior are as follows: (1) a personal financial crisis that cannot be shared, (2) an opportunity to breach trust, and (3) the ability to reconcile the cognitive dissonance associated with violating one’s own ethical standards. Fraud opportunity is perceived to exist when an individual believes that s/he can take advantage of systemic weaknesses such that fraud can be committed with little probability of being caught or punished, and/or when the cost of punishment is not high enough in the event of being caught.

The fraud triangle provides no direct explanation of how opportunities for fraud are perceived. Fraud opportunity can be considered from two perspectives: temptation and obstacles. When an opportunity for fraud presents itself, such as under conditions of weak internal control, a low likelihood of detection, etc., temptation arises and can lead to fraud committal under “imperfect” or “incomplete” conditions. However, obstacles rendering the decision to commit fraud less likely can also arise. For example, the individual may suppress their impulse to commit fraud, and think about the risks of engaging in fraudulent behavior under conditions of strong internal control, which may also cause hesitancy about abusing a privileged position.

### Motivation

Fraud can be explained by both the individual characteristics of the perpetrator and the environment. Bonger (1969) states there must be an environment conducive to crime, where who actually commits crimes depends on individual factors. Bem and Funder (1978) posited that a behavior is a function of the mutual interaction between a subject and their circumstances. To account for human factors in the decision-making process, motivation can be classified in terms of desire and motivation for action; their interaction reflects that between the individual and situation. Desire depends on the innate nature of the individual, and is thus stable and unchanging, while the motivation for action relates more to the environment.

### Disposition

As mentioned above, desire depends on the character and traits of the individual. Disposition underpins the differences among people (Raval, 2018). Setiya (2007) posits that disposition is a virtue framed in a practical way. According to Tilak (2004), disposition reflects the inner nature and can be divided into three categories: enlightened, passionate and indolent. These different categories, and the degree to which they are expressed, make people different from each other. Disposition reflects faith or confidence, which acts as a driving force in one’s choices. The definition of disposition in the Oxford Dictionary is “a person’s inherent qualities of mind and character.” Disposition is inherent, and is thus independent of (and unaffected by) the external environment. It is stable and persistent, being fixed across situational contexts. Buss and Craik (1983) described disposition in terms of reliable and consistent behavior. Katz (1993) referred to disposition as a habit of mind expressed frequently in a conscious and intentional manner. A thorough review of the literature on conceptions of disposition revealed that one of the most popular Hindu scriptures classifies disposition into three types: (1) passionate and able to tell right from wrong; (2) passionate but not able to tell right from wrong; and (3) lacking passion and showing ignorance. Among these dispositions, this paper focuses on the first two types, as individuals without passion will not show interest or enthusiasm for performing the actions of interest herein. Individuals in the first two disposition categories both have passion and a desire to act. However, people with passion and knowledge are more often faced with an inner battle between morality and emotional impulses, and will thus tend to “think twice” before taking action, while people with passion but no knowledge are driven by impulses to act instantly and without prudence. Green (1906) divides disposition into self- and other-regarding types. Self-regarding describes people who focus on their own interests and have no consideration for others, which corresponds to the lowest three of the six stages of moral hierarchy: social norms, self-interest orientation, and obedience and punishment orientation (Kohlberg, 1984). Meanwhile, other-regarding describes people exhibiting unselfish devotion to others and aligns with the highest three stages of the moral hierarchy: law and order morality, social contract orientation, and universal ethical principles. In summary, people who are other-regarding and can tell right from wrong will experience more inner struggle before deciding to commit fraud, while people who are self-regarding and reckless are likely to succumb to the temptation of fraud. Based on these considerations, we propose the following hypothesis:

*H1*: People with high levels of self-regard are more likely to commit fraud.

### Self-control

Financial fraud can be conceived of as an act of indulgence; when opportunities for fraud exist, such as under conditions of weak internal control, temptation arises. Self-control refers to the ability to restrain oneself in the face of temptation. As mentioned above, self-regarding people are vulnerable to temptation, but self-control acts as a counterforce. According to Mischel (2014), self-control may reduce the dispositional tendency to commit immoral behavior. Against this background, we propose a second hypothesis:

*H2*: Self-control negatively moderates the positive correlation between self-regard and the tendency to commit fraud.

### Self-efficacy

According to the definition of the American Psychological Association (2009), self-efficacy reflects confidence in the ability to complete a task or goal. It is a cognitive self-evaluation that can affect future behavior and is based on one’s past experiences. During the process of deciding whether to commit fraud, the individual has to overcome barriers such as strong internal control, under which few opportunities for fraud arise. In addition, the individual will also try to avoid being caught when under strict supervision. Wolfe and Hermanson (2004) added capability to the fraud triangle, positing that the individual’s ability to commit fraud is also a key determinant of actual fraudulent behavior. Different from capability or ability, self-efficacy relates more to the perception and will to carry out a behavior. People with high levels of self-efficacy have more confidence in their ability to overcome obstacles and are more likely to grasp a rare opportunity and fulfill their intention. According to this perspective, we propose a third hypotheses:

*H3*: Self-efficacy moderates the positive correlation between self-regard and the tendency to commit fraud.

## Methodology and data collection

### Methodology

**Figure 1:**
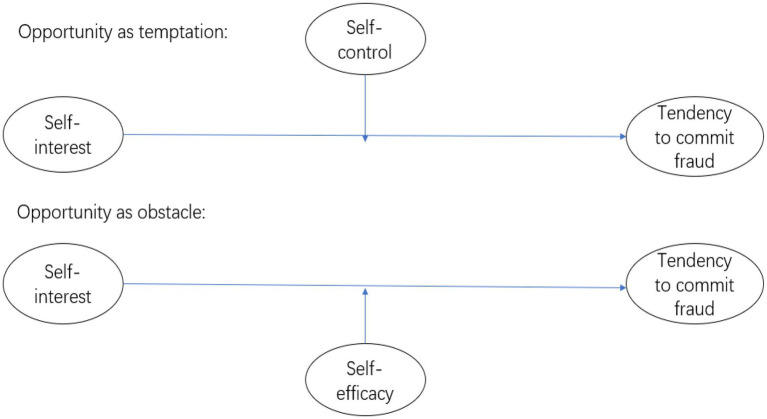
The research framework.

As shown in Figure 1, the conceptual framework of this research is based on the above-described hypotheses.

### Design of the questionnaire and data processing

A multi-dimensional measurement questionnaire was designed. Each question was based on existing, verified scales. The nine questions on self-interest, which in this study is taken to be a proxy of self-regard, were based on the scale of Gerbasi and Prentice (2013). The 12 items on self-control were derived by omitting items pertaining to unrelated constructs, such as physical activities, the performance of simple tasks, as well as repeated constructs, such as self-interest and self-centeredness, from the scale developed by Grasmick et al. (1993). The 10 questions on self-efficacy were based on Schwarzer and Jerusalem (1995). The fraud tendency questions were based on the scenario constructed by Ng et al. (2009).

A 7-point Likert scale was for the responses, with 7 corresponding to *strongly agree* and 1 to *strongly disagree*. All data collected through the questionaries were then centralized to prevent multicollinearity between the independent and moderator variables, and to allow for the testing of interaction effects. After the centralization, the sum of scores given to all questionnaire items would be zero after deducting the average. The following mathematical equation depicts the “centralization” process:


$$\sum \left(X_{i}-\bar{x}\right)=\sum Y_{i}=0$$

### Sampling method and data analysis

This study enrolled people with accounting or financial work experience. Fifty questionnaires were distributed to experts for a pilot study. After revising the questionnaire based on the pilot, we distributed 600 questionnaires in the main study; 532 were returned (response rate of 88.67%).

**Figure 2:**
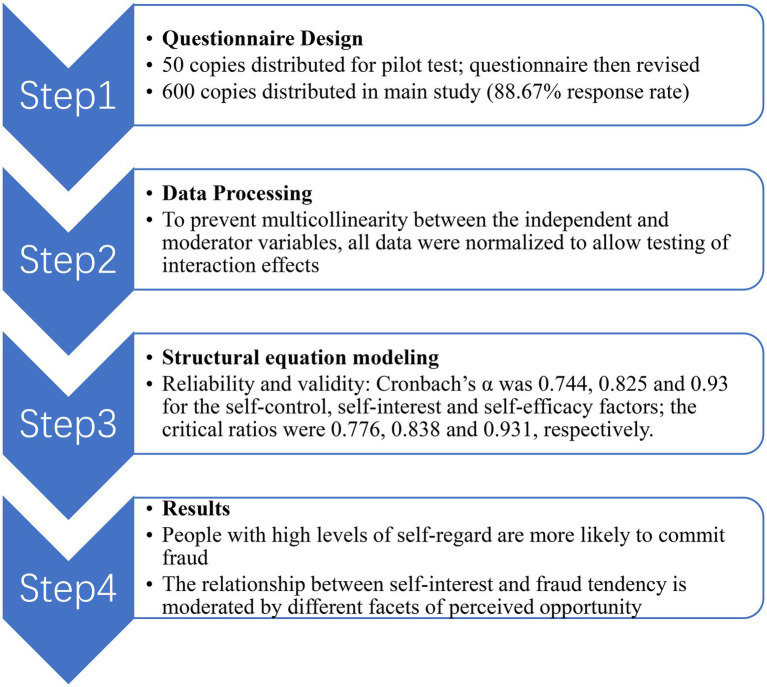
Methodological approach.

This research applied linear structural equation modeling (SEM) and confirmatory factor analysis (CFA) to analyze the data and validate the research framework. AMOS 25.0 software (SPSS Inc., Chicago, IL, United States) was used to perform the CFA. The questionnaire has three implicit factors (Self-interest, Self-control, and Self-efficacy), each comprising observable/explicit variables (listed below). The survey was based on these observable/explicit variables; multiple questionnaire items were classified under each of them. Figure 2 shows the methodological process and Table 1 displays the questionnaire items for each implicit/explicit variable, as well as the corresponding references. Since each item in the questionnaire is come from mature scales, hence we employed confirmatory factor analysis (CFA) to verify the reliability of the matures scales.

**Table 1:** Questionnaire items under Implicit Variables and Observable Variables.

Implicit variables	Explicit variables	Number of items	Associated reference
Self-interest		9	Gerbasi and Prentice (2013)
Self-control		12	Grasmick et al. (1993)
Self-efficacy		10	Schwarzer and Jerusalem (1995)
	Fraud tendency	4	Ng et al. (2009)

### Sample description

The proportions of male and female respondents were 52.82 and 47.18%, respectively. The proportion of respondents aged 18–25 years was 10.71%, compared to 10.73% for those aged 26–30 years, 60.71% for those aged 31–40 years, 10.71% for those aged 41–50 years, and 7.14% for those aged 51–60 years. The proportion of respondents with less than 5 years of work experience was 21.43%, compared to 46.43% with at least 5 years but less than 10 years, 21.43% with at least 10 years but less than 20 years, and 10.71% with at least 20 years. The education level distribution was as follows: bachelor degree, 21.43%; master degree, 78.57%.

## Results

SEM was used to analyze the data and test the hypotheses proposed above.

### Reliability and validity of the results

As shown in Table 2, the Cronbach’s α, which is used to measure reliability, was 0.744, 0.825 and 0.93 for the self-control, self-interest, and self-efficacy factors of the questionnaire, respectively. The critical ratio (CR) was 0.776, 0.838 and 0.931 respectively; thus, the questionnaire is highly reliable.

**Table 2:** Factor loadings and reliability of the questionnaire items.

	Factor loading	α	AVE	CR
I1	0.679	0.825	0.683	0.838
I2	0.751			
I3	0.697			
I4	0.781			
I5	0.845			
I6	0.539			
I7	0.587			
I8	0.658			
I9	0.526			
C1	0.568	0.744	0.686	0.776
C2	0.526			
C3	0.612			
C4	0.596			
C9	0.658			
C10	0.913			
C11	0.655			
C12	0.817			
C21	0.658			
C22	0.594			
C23	0.614			
C24	0.604			
F1	0.673	0.93	0.576	0.931
F2	0.880			
F3	0.700			
F4	0.847			
F5	0.737			
F6	0.788			
F7	0.812			
F8	0.747			
F9	0.713			
F10	0.661			

“I,” self-interest; “C,” self-control; “F,” self-efficacy.

As shown in Table 2, the average variance extracted (AVE) values were all above 0.5, as were the factor loadings, indicating good convergent validity.

As Table 3 shows, the square root of AVE exceeded the correlation coefficient between pairwise variables in all cases. Thus, the questionnaire shows good discriminant validity.

**Table 3:** Mean and standard variance of the questionnaire factors.

	Mean	Standard variance	I	C	F
I	4.58	1.35	**0.826**	0.419^**^	0.530^**^
C	3.82	0.73	0.419^**^	**0.828**	0.141^**^
F	5.18	0.85	0.530^**^	0.141^**^	**0.759**

“I,” self-interest; “C,” self-control; “F,” self-efficacy. The bold number on the diagonal is the square root of AVE; the number below or above the diagonal is the correlation coefficient. **p < 0.01 (two-tailed test).

**Figure 3:**
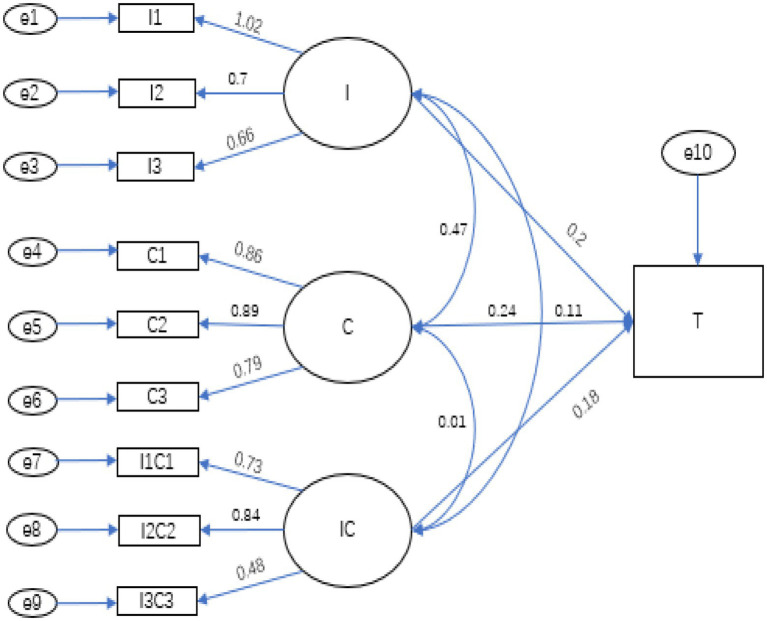
Pathways from opportunity (giving rise to temptation) to fraudulent behavior. “T,” fraud tendency.

**Figure 4:**
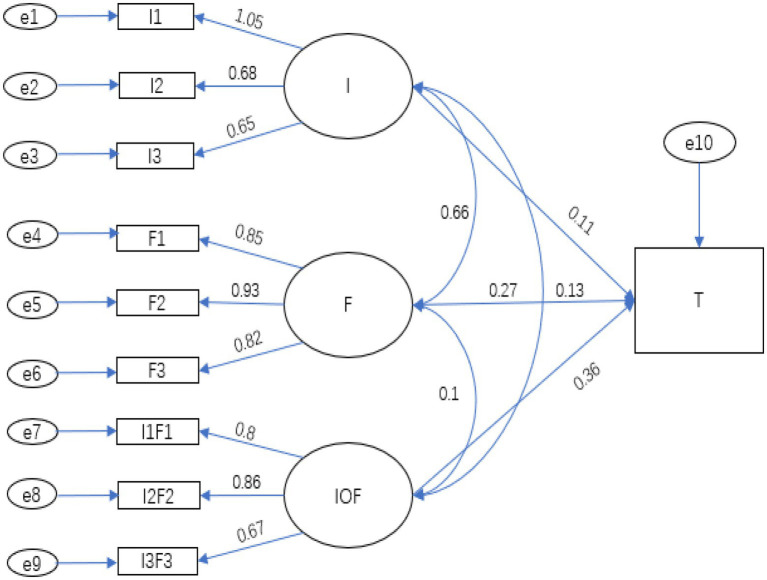
Pathways from opportunity (perceived as an obstacle) to fraudulent behavior.

SEM was used to test the hypotheses proposed above. First, we centralized all independent and moderator variables; second, the factor loadings of the independent and moderator variables were sorted from high to low, and grouped according to the principle of “large with large, small with small” to produce the product index after centralization; Finally, the independent variables, moderator variables and newly generated paired product index were added to the model for the analysis. The fitting results are shown in Figures 3, 4, as well as Tables 4, 5.

**Table 4:** Pathways from self-interest to tendency to commit fraud corresponding to Figure 3.

	Estimate	Standard estimate	S.E.	CR	P
I → T	0.407	0.204	0.098	4.143	<0.001
C → T	0.158	0.113	0.071	2.237	0.025
IC → T	0.365	0.184	0.100	3.661	<0.001

CMIN/DF = 2.247; RMSEA = 0.069; CFI = 0.925; IFI = 0.926; GFI = 0.961.

**Table 5:** Pathways from self-interest to tendency to commit fraud corresponding to Figure 4.

	Estimate	Standard estimate	S.E.	CR	P
I → T	0.225	0.11	0.106	2.112	0.035
F → T	0.173	0.125	0.077	2.242	0.025
IOF → T	0.483	0.356	0.064	7.512	<0.001

CMIN/DF = 2.649; RMSEA = 0.042; CFI = 0.954; IFI = 0.955; GFI = 0.929.

As we can see from Table 4, the model passed the goodness-of-fit test; that is, self-interest (I) and tendency to commit fraud (T) had a significantly positive relationship, and self-control (C) acted as a significant positive moderator of that relationship. Thus, H1 and H2 are supported.

As we can see from Table 5, the model passed the goodness-of-fit test; that is, self-interest (I) and tendency to commit fraud (T) had a significantly positive relationship, and self-efficacy (F) acted as a significantly positive moderator of that relationship. Thus, H3 is supported.

## Discussion and conclusion

From the results presented above, we derived the following conclusions:

1. The questionnaires developed in this study had satisfactory reliability and validity, based on the results of SEM.
2. People with high levels of self-regard are more likely to commit fraud (Hypothesis 1 is supported). When an opportunity for fraud, i.e., temptation, arises, a low level of self-control significantly increases the tendency to commit fraud (Hypothesis 2 is supported). Meanwhile, when an opportunity for fraud is perceived as an obstacle, a high level of self-efficacy significantly moderates the tendency to commit fraud (Hypothesis 3 is supported).

### Contributions of this study

Unlike previous studies of financial fraud motivation that either completely changed the triangle fraud theory or made modifications to the classical model, this study provides explanations of psychological perceptions in given environments based on the original triangle model. Therefore, this study better explains the influence of human factors on fraudulent behavior. Moreover, unlike other studies focusing on eliminating the opportunity for fraud, such as by perfecting internal control or applying strict supervision and severe punishment (which is unrealistic in the real world), this study focuses more on human cognitions and innovatively explores human perceptions of the opportunity for fraud; pathways to final behavioral decisions are elucidated. The influence of psychological factors on fraudulent behavior merits further research in relevant fields, to potentially improve the effectiveness of regulation and enhance auditing efficiency and control frameworks.

Another contribution of this study was the application of innovative research methods. The literature review showed that previous studies on fraudulent behavior considering human factors are largely qualitive or theoretical. This study applied a quantitative approach for analyzing human factors. Studies on the tendency to commit fraud tend to use multiple regression analysis and seldom adopt the CFA-based research framework, which takes the moderating effects of implicit variables into consideration. CFA and linear SEM were deemed suitable for this study, given that the main variables of interest were implicit. As such, the innovative methodology used in this study can be considered valid.

### Limitations and suggestions for future research

Regarding study limitations, there are general concerns regarding survey data, where for example respondents might not always answer truthfully. However, given that our survey was anonymous, we believe this problem was not a significant factor in this study. We were also concerned that the respondents might fill in the first and/or second sections of the survey, but leave the last section blank. If this were the case, we would expect to see a higher proportion of respondents answering the questions appearing at the beginning of the survey. However, the response rate did not differ between the questions at the beginning and the end of the survey.

Finally, human psychology is influenced by a large number of complex factors, but this study focused only on factors such as self-control and self-efficacy; thus, some factors influencing the decision-making process were likely not analyzed. Future studies could consider extending the scope of the research by analyzing additional factors influencing human psychology, to obtain a more detailed picture of the decision-making process.

## Data availability statement

The raw data supporting the conclusions of this article will be made available by the authors, without undue reservation.

## Ethics statement

The studies involving human participants were reviewed and approved by Dongbei University of Finance and Economics. The patients/participants provided their written informed consent to participate in this study.

## Author contributions

XS: conception and design of study, acquisition, analysis, and interpretation of data, and drafting the manuscript. YC: revising the manuscript critically for important intellectual content and approval of the version of the manuscript to be published. All authors contributed to the article and approved the submitted version.

## Conflict of interest

The authors declare that the research was conducted in the absence of any commercial or financial relationships that could be construed as a potential conflict of interest.

## Publisher’s note

All claims expressed in this article are solely those of the authors and do not necessarily represent those of their affiliated organizations, or those of the publisher, the editors and the reviewers. Any product that may be evaluated in this article, or claim that may be made by its manufacturer, is not guaranteed or endorsed by the publisher.
